# Robot-Based Calibration Procedure for Graphene Electronic Skin

**DOI:** 10.3390/s22166122

**Published:** 2022-08-16

**Authors:** Jan Klimaszewski, Krzysztof Wildner, Anna Ostaszewska-Liżewska, Michał Władziński, Jakub Możaryn

**Affiliations:** 1 Warsaw University of Technology, Faculty of Mechatronics, Institute of Automatic Control and Robotics, A. Boboli 8 Street, 02-525 Warsaw, Poland; 2 Warsaw University of Technology, Faculty of Mechatronics, Institute of Metrology and Biomedical Engineering, A. Boboli 8 Street, 02-525 Warsaw, Poland

**Keywords:** electronic skin, calibration, human–machine interface

## Abstract

The paper describes the semi-automatised calibration procedure of an electronic skin comprising screen-printed graphene-based sensors intended to be used for robotic applications. The variability of sensitivity and load characteristics among sensors makes the practical use of the e-skin extremely difficult. As the number of active elements forming the e-skin increases, this problem becomes more significant. The article describes the calibration procedure of multiple e-skin array sensors whose parameters are not homogeneous. We describe how an industrial robot equipped with a reference force sensor can be used to automatise the e-skin calibration procedure. The proposed methodology facilitates, speeds up, and increases the repeatability of the e-skin calibration. Finally, for the chosen example of a nonhomogeneous sensor matrix, we provide details of the data preprocessing, the sensor modelling process, and a discussion of the obtained results.

## 1. Introduction

The development of electronic skin (e-skin) technologies is still a current research topic, undertaken by scientific teams worldwide for over three decades [1]. The concept of an extensive, flexible network of sensors as an interface that mimics a biological organ is being developed, particularly in medicine, to enable smart healthcare [2], prosthesis [3], and amplifying human sensory abilities [4,5]. Apart from medicine, the second area of particularly dynamic development of the e-skin concept is robotics [6], where one of the research trends is to improve the safety of human–machine cooperation and the agility of robotic manipulation [7,8,9,10]. Accurate sensing of the touch force is an important issue in soft robotics, where it is used for delicate interaction with the environment in flexible grippers [11], which might be equipped with additional sensors for magnetic field [12], temperature [13], or humidity and proximity [14].

In recent years, much research has been carried out worldwide on pressure-measuring flexible devices. These studies have included, among others, estimating the value and location of the touch pressure. The subject of research is also the development of technology for producing flexible printed electronics that have taken place over the years [15]. The application and classification of operating methods for pressure-measuring devices are also analysed [16,17]. In recent years, graphene-based e-skin sensors characterised by higher sensitivity compared with other types of sensors have become more available [18,19,20,21]. The dynamic development is also visible in low-cost e-skin production [20,22,23].

As part of the authors’ previous research, a fragment of e-skin has been made based on graphene sensors placed on a flexible foil [20]. It allows to determine the location, value, and direction of touch pressure and to measure the estimated distance of a part of the human body [24] in e-skin proximity. The data acquisition time for touch pressure from 32 × 16 sensors is approximately 21 ms, and proximity estimation can take as little as a few μs. An additional advantage of the developed e-skin is its low fabrication cost.

Many authors try to use e-skin to calibrate and self-calibrate the kinematic chain for rigid mechanisms [25,26,27,28] as well as to measure the deformation and position of soft robots [29]. An attractive solution is to supplement the calibration process with data from the stereovision camera described in [27]. Other authors use touch sensors, e.g., for 3D reconstruction of the surface touched by the manipulator [30]. In these publications, it is worth paying particular attention to the modular structure of the skin and the use of information about the gravity vector for each module. Another important issue is diagnostics of electronic skin, when it is used as a safe interface for human–robot cooperation. The high variability of parameters between sensors, especially the low cost, makes the practical use of e-skin extremely difficult. As the number of sensors in e-skin increases, this problem worsens. Therefore, solving the problem of calibrating many e-skin matrix sensors with nonuniformity of their parameters [3] is of great importance in the currently conducted development works. In terms of calibrating and establishing the measurement characteristics of e-skin devices, a standard approach to solving this problem can be found in the literature. Researchers typically construct a test stand to apply precise pressure to one selected e-skin sensor, allowing simultaneous measurement of this pressure with a reference device. The simplest approach can be found in [31], where e-skin touch calibration was performed based on a reference in the form of weights of known mass manually placed at selected locations on the e-skin surface. In [32], an XY table and a voice coil were used to exert the force. Linear, quadratic, and cubic polynomial models were fitted to data to calibrate the example sensor. The best results were obtained using a Huber regression with a quadratic polynomial model. In [33], a reference load cell was fixed on the z-axis platform to apply pressure to the e-skin’s individual sensors. In [34], no direct calibration of the pressure value was carried out—only the relationship between the variable capacitance and the measured voltage value for capacitance-based e-skin. As a result, a fourth degree polynomial model was used as calibration curve. A high-precision pressure testing platform was used in [35]. In each of the cases described, the parameters of the various e-skin sensors were assumed to be homogeneous. The calibration procedures presented would be labour-intensive and difficult to repeat in practice for a large number of heterogeneous e-skin sensors. No publication addressing this issue was found in the literature reviewed.

The primary aim of the presented research is to propose a semiautomated calibration procedure for a distributed electronic skin built based on graphene FSR sensors with nonhomogeneous parameters. We describe the robot-based test setup and the calibration procedure together with the measurement data analysis, which allows for calibration procedure automation and precise measurement of the force exerted on the e-skin sensors. Then, a method is proposed for determining a family of measurement characteristics of touch sensors and studying the variation of their parameters on the surface of e-skin. It allows for the understanding of the properties of manufactured e-skin. In addition, the developed procedure allows running e-skin diagnostics—detecting its erroneous and correctly functioning elements.

The paper is organised as follows. Section 2 presents an overview of the methods, devices, and models used in the manuscript. In particular, the e-skin description, calibration setup, and calibration procedure are discussed. In Section 3, the calibration results are presented; this section details the initial stage of data preprocessing and preparation, the modelling process of the e-skin sensors, and a discussion of the results obtained. Finally, in Section 4, the conclusions are given.

## 2. Methods and Models

The manuscript describes the procedure for e-skin calibration. For this purpose, a test stand was built, where the measurements necessary to perform the calibration were acquired. Once the data acquisition process was complete, the measurements were analysed. The aim was to determine the characteristics describing the relationship between the e-skin sensors’ touch pressure and the force exerted on the e-skin. The next section introduces the e-skin to be calibrated (Section 2.1); describes the prepared calibration stand (Section 2.2); and finally, presents the calibration procedure (Section 2.3).

### 2.1. Electronic Skin Description

The electronic skin used in the research allows to determination of the position and force exerted by the object on the surface on which it was placed. The e-skin system is made of a matrix of force-sensing resistors (FSR). Only the key information on e-skin necessary to present the calibration issue is described below. Extensive information on the details of e-skin construction has already been presented in the literature [20].

**Figure 1:**
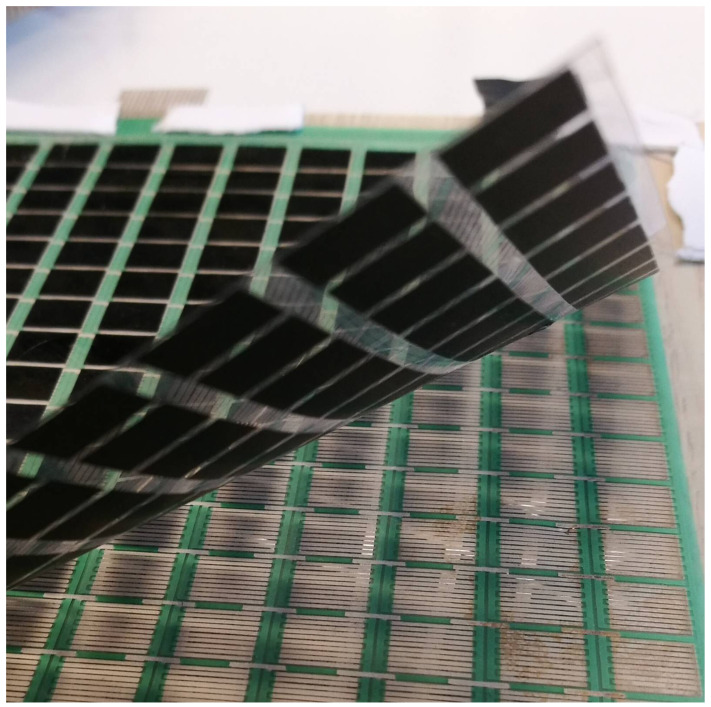
The robotic skin: the upper FSR sensors arranged in a rectangular pattern placed on a plastic foil, and the lower conductive layer of comb electrodes printed on plastic foil.

The sensor matrix is made of two layers of plastic foil (Figure 1). The comb electrodes are placed on the bottom layer. The electrodes and the pathways connecting them are made with silver-based paint. The top layer has graphene FSR fields placed directly above the comb electrodes. Each of the FSR fields is approximately 5 mm × 5 mm in size and manufactured using graphene nanoplatelets in a similar way to those known from bibliography [36]. The entire sensor matrix comprises 512 sensors organised into 16 rows and 32 columns. Each comb electrode placed under the graphene FSR field comprises two parts. One part is connected to all the electrodes in a row and the other to all the electrodes in a column. The pressure of the graphene field on the electrodes makes them conductive. By measuring the resistance between the selected rows and columns, the location can be determined and the value of the touch pressure exerted on the FSR matrix can be estimated.

**Figure 2:**
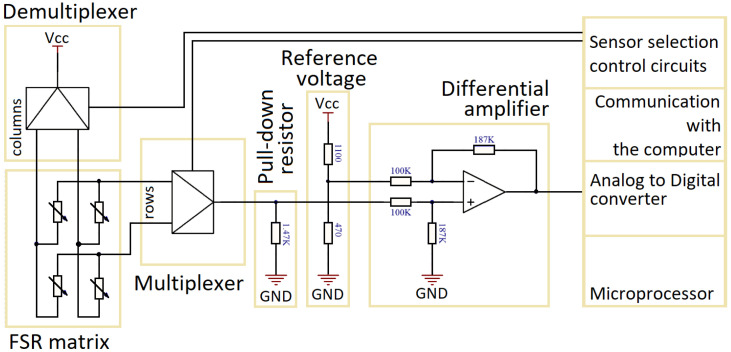
Block diagram of the electronic system for determining the localisation and a value of touch pressure exerted on the FSR matrix.

A dedicated electronic system has been made for control and measurement of touch pressure (Figure 2). In the developed system, the matrix of sensors is connected via an analog demultiplexer connecting the supply voltage to the selected column. An analog multiplexer connected to each row allows to select a single FSR resistance measurement. A pull-down resistor connects the FSR to the ground, allowing the test current to flow through it. The difference between the voltage across the pull-down resistor and the reference voltage is amplified and fed to the analog–digital converter.

**Figure 3:**
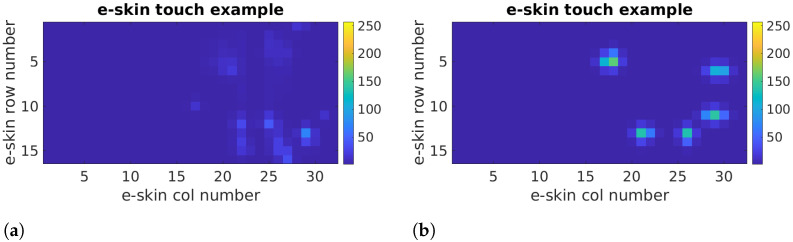
Example hand touch visualisation for the size of 16 × 32 cells; Gaussian blur used for postprocessing. (**a**) Example hand touch. (**b**) Example fingertips touch.

The e-skin controller measures the pressure for each sensor and then transmits the position and touch pressure exerted on the active surface. The data are sent to a computer where they are processed and saved. The computer software enables the visualisation of the obtained results as a colour-coded image. The images (Figure 3) show the pressure on the fingers and the pressure of the entire hand exerted on the e-skin.

### 2.2. Calibration Stand

E-skin calibration aims to define a family of measurement characteristics of touch sensors placed on the surface of the e-skin. These measurement characteristics should describe the relationship between e-skin touch pressure and the force exerted on the e-skin surface. To perform calibration, it is necessary to collect data from a reference force measuring device and e-skin. The latter may have several dozen to even several hundred resistance force sensors organised as a rectangular matrix. Manual examination and analysis of each sensor’s data is a very tedious and lengthy process. To carry out the automated measurement data acquisition process, the robotic calibration setup and the robotic application were developed.

**Figure 4:**
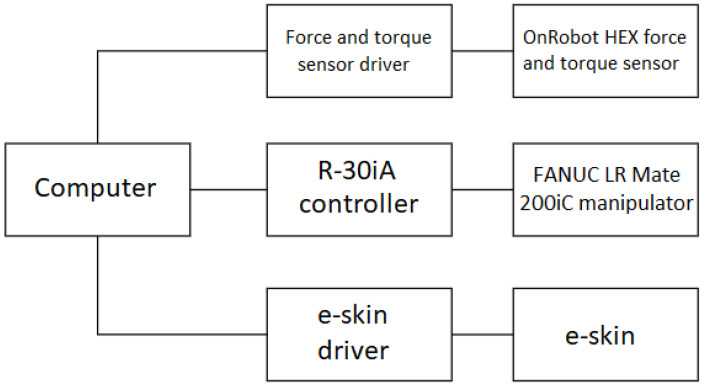
Block diagram of the system used in the measurements. E-skin describes FSR matrix from Figure 2; e-skin driver describes the rest of the electronic system from Figure 2.

The measurement acquisition setup consists of the FANUC LR Mate 200iC manipulator, the R-30iA Mate manipulator controller, the e-skin with a driver, the OnRobot Hex-e 6-axis force and torque measuring device with a controller, and a general purpose PC. The general connection diagram of the calibration setup devices is shown in Figure 4.

The e-skin via the dedicated electronic measuring system was connected to a PC using a USB port and serial communication. The controller connected in this way, using the appropriate application, is ready to send readings from e-skin sensors. Measurement data were saved to a text file.

The Hex-e device is compatible with the Fanuc LR Mate 200iC manipulator wrist. This made it easy to integrate the Hex-e device on the manipulator. A dedicated tool made using a 3D printing technology (FDM) was further bolted to the force sensor. A pressure spring was installed in the manipulator tool construction due to the build-up of force exerted on the e-skin because the displacement of the tool tip is slower. This allows better control of this force by the displacement of the robot tool tip. This is important because graphene-based FSRs are usually highly sensitive to the low force exerted on them. In a situation, as in the designed test setup, where control of the force exerted on the e-skin is indirectly achieved by displacement, it is necessary to significantly slow down the displacement of the tool tip in order to acquire a large number of measuring points for low contact pressures. By lowering the stiffness of the closed kinematic chain created when the tool tip contacts the e-skin surface, a greater number of measurement points can be acquired for a minimum manipulator movement speed than would be the case with a rigid tool. Furthermore, by using a linear velocity of the manipulator movement, it is possible to achieve a linearly increasing force exerted on the e-skin. This can be an important factor in calibration.

To acquire measurements from the Hex-e sensor, a controller provided by the manufacturer was used. The controller was used for the acquisition of measurements and allows them to be transferred to a PC via Ethernet. The FANUC LRMate 200iC manipulator was connected to the R30-iA Mate control cabinet, which communicated with a PC also via Ethernet.

**Figure 5:**
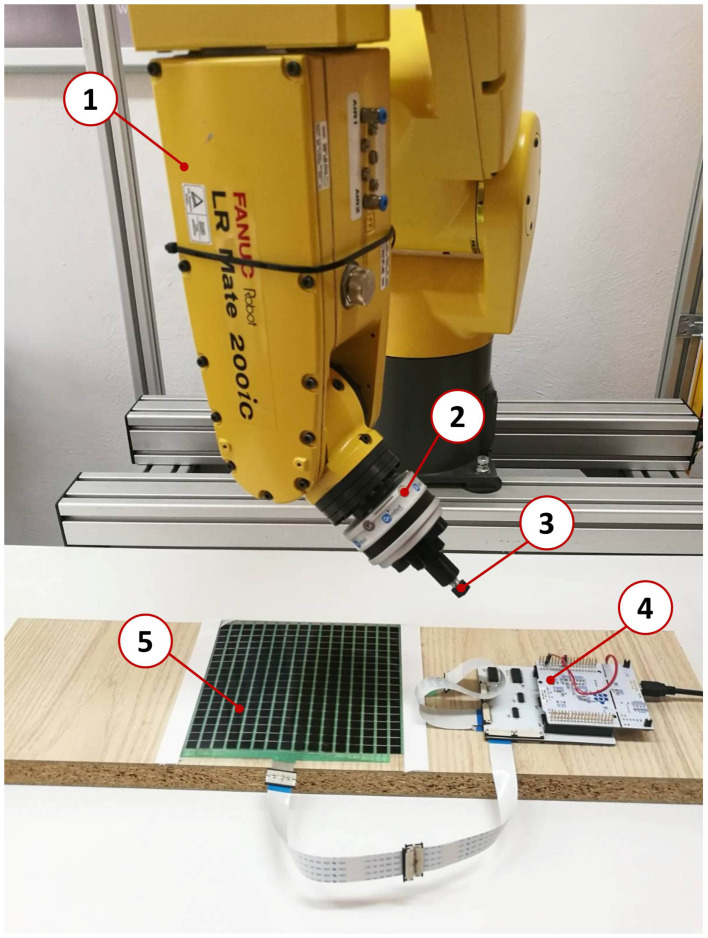
Illustrative representation of the calibration station: 1. LRMate 200iC manipulator, 2. reference e-Hex sensor, 3. robot tool, 4. e-skin electronic system, 5. e-skin.

Figure 5 presents photos of the calibration stand prepared for data acquisition from the e-skin and Hex-e device.

### 2.3. Calibration Procedure

The calibration procedure consisted of two stages. During the first stage, data from e-skin sensors and the reference Hex-e sensor were acquired. The second stage aimed to estimate the relationship between each of the e-skin sensors with the reference Hex-e sensor and, next, to analyse aggregated results of this estimation. Both stages were additionally subdivided into the ‘loading phase’—when the force exerted on the particular sensor of the e-skin by the robotic arm was increasing, and the ‘unloading phase’—when force was decreasing. The data analysis was performed independently for the ‘loading’ and ‘unloading’ phases.

#### 2.3.1. Stage I

Prior to measurements, the coordinate systems of the Fanuc robot were calibrated. The tool frame was calibrated using the standard 3-point method by approaching one stationary point, localised within the robot’s surroundings, three times—each time with a different approach vector. The user frame was calibrated with the standard 3-point method by selecting the system’s origin, a point on the X-axis defining its direction, and a point belonging to the XY plane. The TCP (Tool Centre Point) was set to be in the centre of the tool that exerts pressure on the sensors. The XY axes of the user frame were aligned with the columns and rows of the e-skin sensors, respectively. One of the e-skin corners was indicated as the origin of the user frame.

**Figure 6:**
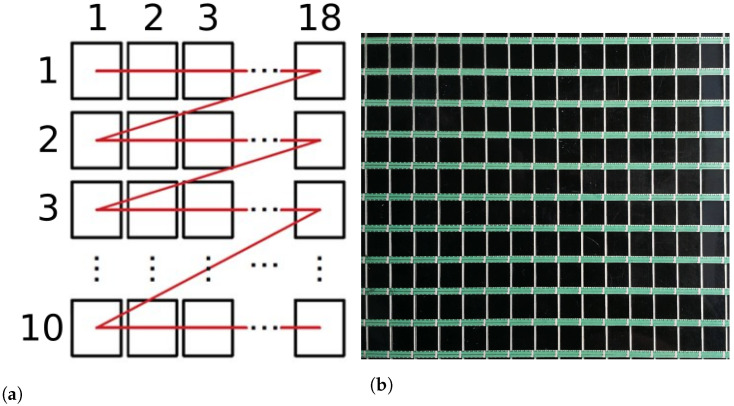
Illustrative presentation of robot trajectory during Stage I of the calibration procedure. (**a**) Robot trajectory path. (**b**) E-skin surface patch covered by robot trajectory.

After the calibration of coordinate frames, the measurements of the e-skin were performed. The industrial robot was programmed to move above the e-skin matrix and sequentially position the tool above each of the e-skin sensors from the subset being analysed (10 rows × 18 columns). Robot trajectory is illustrated in Figure 6. For each sensor, the ‘loading’–‘unloading’ sequence was performed. The program developed for this purpose might be adapted to other e-skin geometries. 

The ‘loading’–‘unloading’ sequence was implemented by lowering the tool slowly so that the tip of the tool pressed the sensor and then withdrew the tool at the same speed, along the same trajectory. The distance the tool should move was chosen experimentally prior to the measurements to cover the e-skin sensors range. No force feedback was implemented.

Data acquired during experiments were stored in 720 text files in total (10 rows × 18 columns × 4 data sets). These files contained measurements from the Hex-e sensor and the e-skin matrix (data and timestamps for the synchronisation), obtained during increasing (loading phase) and decreasing (unloading phase) the force exerted by the robotic arm on each of the e-skin sensors, respectively (4 data sets per sensor being calibrated). Each file contained an appropriate header required for data identification. The transition between recording data associated with the ‘loading’ and ‘unloading’ phase was triggered by the actual robot position.

#### 2.3.2. Stage II

Data processing was performed with Matlab (Mathworks^®^, v.2021a) software. E-skin data are expressed as a decimal representation of 8-bit values, and reference sensor values are expressed in Newtons. First, data were resampled and trimmed to obtain, for each sensor separately, a pair of vectors (one for the Hex-e and one for the e-skin sensor) of the same length and ensure the time coincidence of data points (this procedure is described in detail in Section 3.1.1). Next, data corresponding to each sensor were verified to detect abnormal recordings, which could indicate possible sensor damage or some problems in the data acquisition chain. Sensors recognised as corrupted were excluded from the subsequent processing (Section 3.1.2). Next, datasets corresponding to each of the sensors were narrowed to the range in which a particular e-skin sensor was giving a nonzero reading. It is important to notice that this threshold force was not uniform among the e-skin sensors. Variability of this value is of great importance for the functioning of the e-skin as a whole and is one of the parameters being analysed during this study. Finally, a fitting was performed. All the algorithms and the obtained results of the analysis are described in Section 3.2.

## 3. Results

### 3.1. Measurements Interpolation

To estimate the correlation between the e-skin sensor *p* and the reference Hex-e sensor *F* (regardless of whether the sensor is loaded or unloaded), two data sets are required: $F_{i}\left(t_{Fi}\right),i=1,2,\cdots ,n$ denotes the reference force measurement points, and $p_{j}\left(t_{pj}\right),j=1,2,\cdots ,m$ denotes the e-skin sensor measurement points. Data from *p* and *F* were acquired with different sampling frequencies: approximately 50 S/s for the e-skin sensor and approximately 10 S/s for the Hex-e sensor. There is no guarantee that n=m, $t_{F1}=t_{p1}$ and $t_{Fn}=t_{pm}$. Additionally, it can be estimated that $\Delta t_{F}=t_{Fi}-t_{Fi-1}\approx 100\;\mathrm{ms}$, and $\Delta t_{F}=t_{pj}-t_{pj-1}\approx 21\;\mathrm{ms}$. Before fitting a model equation to data, the interpolation and resampling should be performed so that the number of points and timestamps values of p(t) match those for F(t). The following procedure was used:

1. For measurements $F_{i}\left(t_{Fi}\right)$ i $p_{j}\left(t_{pj}\right)$, select a common time range $t_{k},k=1,2,\cdots ,r$ such that $t_{1}=max\left(t_{F1},tp1\right),t_{r}=min\left(t_{Fn},tpm\right)$, and $\;Deltat_{k}=t_{k}-t_{k-1}=1\;\mathrm{ms}$;
2. Perform a linear interpolation of the values from the $F_{i}$ series and $p_{j}$ for the $t_{k}$ time, as a result of which interpolated data $F_{k}\left(t_{k}\right)$ and $p_{k}\left(t_{k}\right)$ will be obtained;
3. Select a new time interval $\Delta t_{K}=t_{K}-t_{K-1}=floor\left(\frac{r}{n}\right)$, where floor(·) is rounding down to the integer value;
4. As the corresponding values, select from $F_{k}\left(t_{k}\right),p_{k}\left(t_{k}\right)$ only values for the time $t_{K}$, i.e., $F_{K}\left(t_{K}\right),p_{K}\left(t_{K}\right),K=1,2,\cdots ,q$.

Further analysis was performed for the interpolated data $F_{K}\left(t_{K}\right),p_{K}\left(t_{K}\right)$.

#### 3.1.1. Data Validation

**Figure 7:**
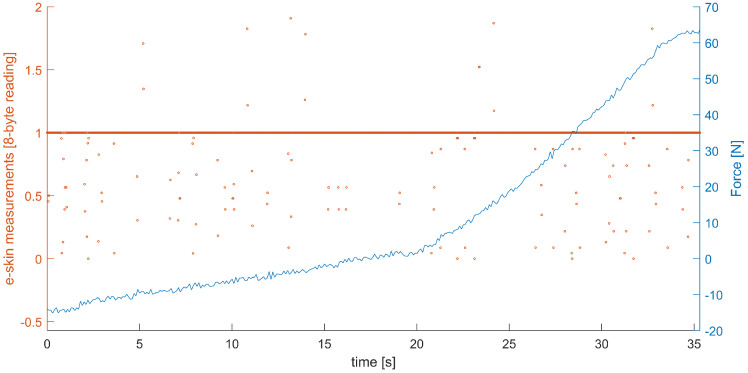
An example of corrupted data: results from the e-skin sensor (orange) are not valid; results from the Hex-e sensor (blue) are correct.

To validate the data recorded from the e-skin and Hex-e sensors, the ratio of the variance *v* and the mean value *m* was calculated (index of dispersion) and assessed. Data were considered as corrupted if the result of |v/m| was less than 3. Such an approach successfully detected 31 malfunctioning sensors—three entire columns of the data subset being analysed and one sensor localised in the edge of the analysed subset of the e-skin. All data classified as corrupted were also verified manually. An example of the corrupted data is shown in Figure 7.

**Figure 8:**
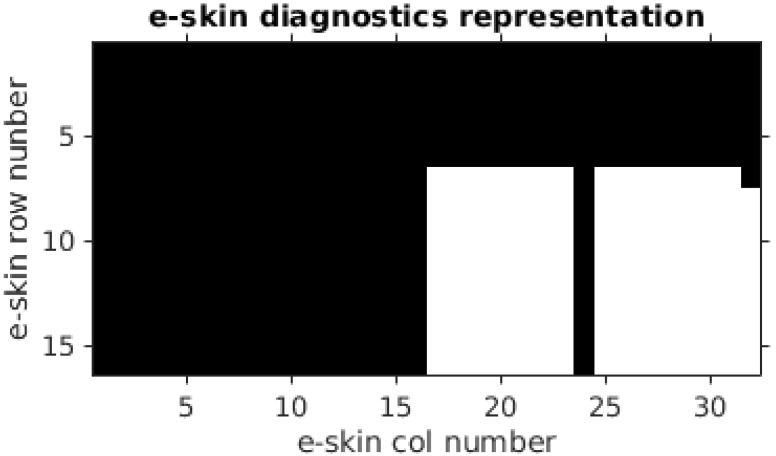
Diagnostic matrix—white squares indicate sensors for which valid data were collected. The e-skin subset being analysed—rows, 7–16; columns, 15–32. Entire columns 15, 16, 24 and the sensors (R7, C32) were found to be not working properly.

As a result of this procedure, a diagnostic matrix (shown in Figure 8) was obtained, which indicated that the e-skin sensors were working properly. It is worth noting that, in order to shorten the study, the calibration procedure involved only a portion (10 rows × 18 columns) of the entire e-skin (16 rows × 32 columns). This excerpt is shown in the bottom-right corner of Figure 8.

#### 3.1.2. Narrowing Down the Scope of Measurement Data

**Figure 9:**
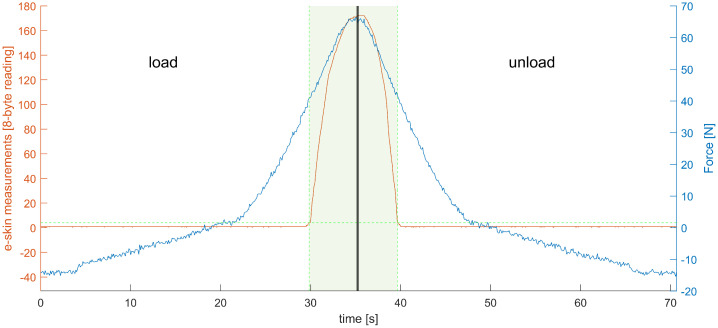
Data recorded while taking measurements from one of the e-skin sensors (row 7, column 19). A thick vertical middle line distinguishes the ‘loading’ phase from the ‘unloading’ phase. The blue line represents the recording from the Hex-e sensor (reference sensor). The orange line represents recordings from the e-skin sensor. The green, horizontal, dashed line is a threshold value based on which narrowing the data range was performed. A data subset used during further processing is indicated by the green shaded area, limited by green, vertical, dashed lines localised in intersections of the threshold line and the e-skin plot.

An example of the recording obtained from one of the e-skin sensors (row 7, column 19) is presented in Figure 9. Both the ‘loading’ and ‘unloading’ phase are shown. It can be observed that there is some threshold value below which results obtained from the e-skin sensor are near-zero.

**Figure 10:**
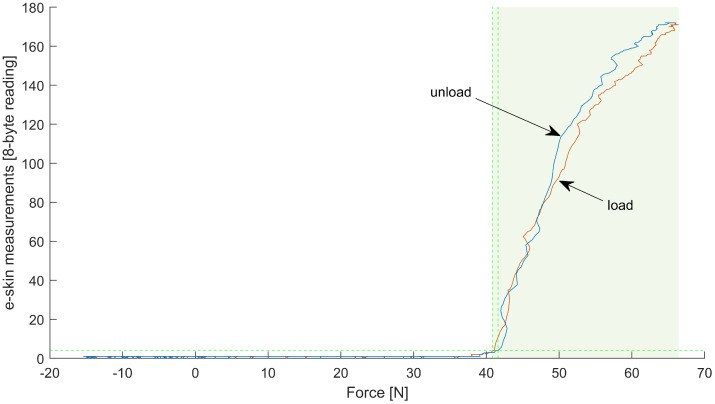
Data recorded while taking measurements from one of the e-skin sensors (row 7, column 19)—data obtained from the e-skin sensor (abscissa) are plotted against data obtained from the Hex-e sensor (ordinate). Data acquired during the ‘loading’ phase are marked orange, and data acquired during the ‘unloading’ phase are marked blue. The green, horizontal, dashed line is a threshold value based on which narrowing the data range was performed. A data subset used during further processing is indicated by a green shaded area, limited by green, vertical, dashed lines localised in intersections of the threshold line and the e-skin plot.

The same can be observed in Figure 10 even more clearly.

It is possible to fit the calibration equation to raw data, without trimming (e.g., logistic function gives the $R^{2}$ at the level of 0.98). The fitting in the range where the e-skin sensor is most sensitive is not as good as it could be if only this subset of data was analysed, however. Thus, prior to the fitting, all data were narrowed to the range in which the particular e-skin sensors are sensitive. This was obtained by means of simple thresholding. It could be observed in Figure 9 that there is around a 2 LSB (the least significant bit) jitter of the zero line. A value of 4LSB was used as the threshold for e-skin data and a value of 3 N as a threshold for HEX data—both thresholds had to be passed. An example of the results of such an approach can be observed in Figure 9 and Figure 10. It can be observed that the force exerted on the e-skin sensor by the robotic arm, corresponding to the threshold level, is around 40 N for this sensor. This force threshold is not consistent for all the e-skin sensors, however. The variability of this parameter influences the sensitivity of the e-skin as a whole. It also makes it difficult to predict the e-skin behaviour at the low level of force applied.

### 3.2. Modelling

**Figure 11:**
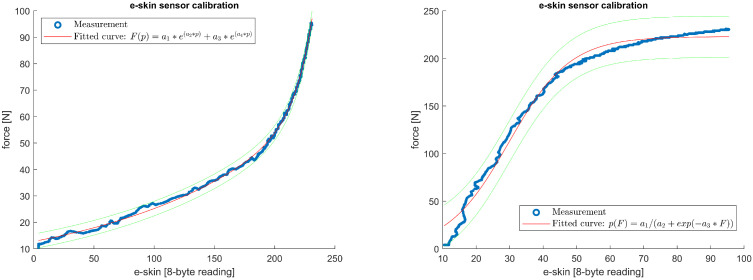
Example plot of curve fitting for measurements in the loading phase of the e-skin sensor from row 15, column 29. On the left is the fit according to Formula (1) and on the right according to Formula (2). Green lines indicate the 95% confidence interval.

**Figure 12:**
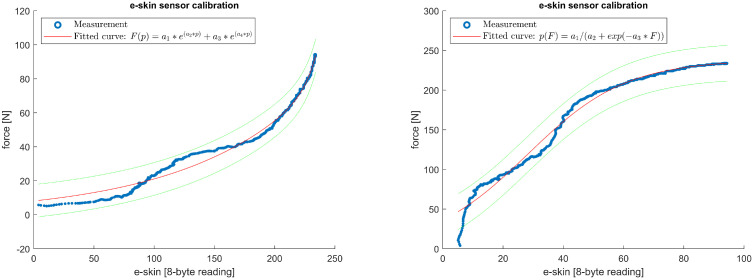
Example plot of curve fitting for measurements in the unloading phase of the e-skin sensor from row 14, column 25. On the left is the fit according to Formula (1) and on the right according to Formula (2). Green lines indicate the 95% confidence interval.

For each of the e-skin sensors, calibration characteristics were obtained by fitting the model equation relating the e-skin sensors’ data with the reference force sensor. The fitting procedure was performed separately for the ‘loading’ and ‘unloading’ phases for all sensors from the analysed subset of the e-skin that passed the validation. Several curve families were considered, from which two were chosen to be used in the calibration procedure: second-order exponential function (Equation (1)) and logistic function (Equation (2)). To assess fitting results, two parameters were analysed: adjusted coefficient of determination (ARS) and root-mean-square error (RMSE). Most of the e-skin/Hex-e characteristics were similar in shape to the one presented in Figure 11 and were well-fitted with the abovementioned equations. For some of the e-skin sensors, however, especially those with a high threshold force, the fitting results were less accurate (Figure 12).

(1) $$F\left(p\right)=a_{1}*e^{a_{2}*p}+a_{3}*e^{a_{4}*p},$$

(2) $$p\left(F\right)=\frac{a_{1}}{1+e^{-a_{2}*\left(F-a_{3}\right)}},$$

where

*F*—force exerted on the e-skin surface in Newtons;
*p*—touch pressure measured by e-skin sensor expressed as a decimal representation of 8-bit values;
$a_{1},a_{2},a_{3},a_{4}$—parameters of individual sensor model curve; each sensor and each curve model (1) or (2) can have different parameters.

Figure 11 (left) shows a plot of the relationship described by Equation (1) in the loading phase of the e-skin sensor from row 15, column 29. As a result of the fitting, the parameters $a_{1}=12.7853,a_{2}=0.0068,a_{3}=3.5153\times 10^{-6},a_{4}=0.0698$ were determined with the fit-quality indicators ARS=0.9989,RMSE=0.8916. For comparison, Figure 11 on the right shows a plot of the curve fitting with Equation (2). The fitting resulted in the determination of the parameters $a_{1}=222.8859,a_{2}=0.1092,a_{3}=29.6893$ with fitting quality factors ARS=0.9883,RMSE=7.7217.

Similarly, Figure 12 (left) shows a plot of the relationship described by Equation (1) in the unloading phase of the e-skin sensor from row 14, column 25. As a result of the fitting, the parameters $a_{1}=8.1001,a_{2}=0.0096,a_{3}=1.6882\times 10^{-10},a_{4}=0.1084$ were determined with the fit quality indicators ARS=0.9875,RMSE=3.1066. For comparison, Figure 12 on the right shows a plot of the curve fitting with Equation (2). The fitting resulted in the determination of the parameters $a_{1}=237.9448,a_{2}=0.0610,a_{3}=28.1509$ with fitting quality factors ARS=0.9846,RMSE=8.0079.

To compare fitting results obtained with both equations, the mean values and standard deviations of ARS and RMSE for all 149 sensors being analysed were calculated (Table 1).

The mean value and standard deviation of each of the coefficients of the fitting curves were calculated to assess their area of variability (Table 2), which might be of interest while performing fitting for other sets of e-skin sensors.

### 3.3. Discussion

**Figure 13:**
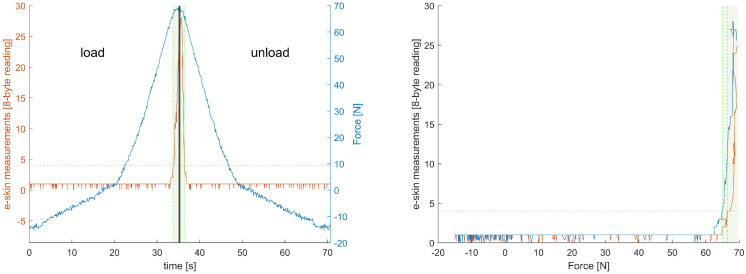
Data recorded while taking measurements from one of the e-skin sensors (row 7, column 26). The markings on the left graph are analogous to those in Figure 9 and on the right graph are analogous to those in Figure 10.

**Figure 14:**
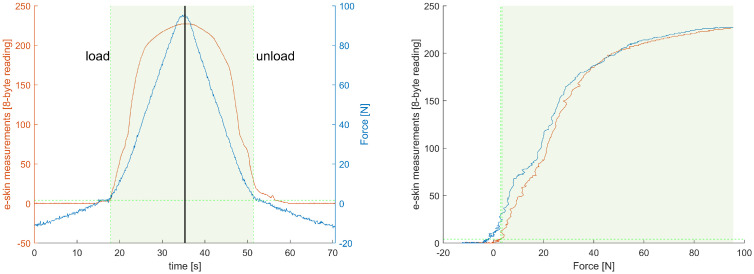
Data recorded while taking measurements from one of the e-skin sensors (row 16, column 30). The markings on the left graph are analogous to those in Figure 9 and on the right graph are analogous to those in Figure 10.

Analysing the raw sample calibration data presented in Figure 13 and Figure 14 allows us to formulate the following observations.

Despite roughly the same force exerted on each e-skin sensor, the touch pressure measured by each sensor differs significantly. This phenomenon may be because of the different parameters of the sensors. Other reasons may include the inaccuracy of defining the robot coordinate frames or an error resulting from the poor repeatability of the robot’s movement—although the latter is less likely. Besides heterogeneous sensor parameters, a reduction in the contact area between the robot tool tip and the e-skin surfaces may also cause differences in sensor measurements. The lack of parallelism of these surfaces may have led to a reduction in the effective contact area, which has a direct impact on the measurement returned by the e-skin sensors.

Analysing further the data shown, for example, in Figure 13 and Figure 14, it can be observed that the force measured by the reference Hex-e sensor passes through the zero point 0 N at a certain moment in time. This is due to the measured gravitational force exerted on the robot tool even before the tool tip contacts the e-skin surface. We can see the effect in both the load and unload phases.

The graphs show that the e-skin sensors have a certain insensitivity threshold for low-contact-force values but are generally better at representing low touch pressure as there is a saturation effect at relatively high values. That insensitivity threshold $F_{in}$, varies widely for different e-skin sensors. This observation was confirmed during the analysis, leading to the modelling of the relationship between force *F* and touch pressure *p*, as shown by the high STD values in Table 3. For example, for the sensor from row 7 and column 26, the threshold $F_{min}$ is around 65 N, while for the sensor from row 16 and column 30, the threshold is around 6.5 N.

For the two example sensors, the range values of returned touch pressure $p_{min},p_{max}$ also differ significantly from each other, whereas the value of the maximum force $F_{max}$ in the two cases does not differ so much. The range of values $p_{min},p_{max}$ for the sensor from row 7 and column 26 changes from 0 to about 30, while for the sensor from row 16 and column 30, the range of these values is from 0 to about 225.

The parameters $F_{in},p_{min},p_{max}$, and others may be affected by factors similar to those discussed in the first paragraph of the current discussion, with the greatest influence on the value of the maximum force $F_{max}$ probably coming from inaccuracies because of the calibration of the robot’s coordinate frames.

It is worth noting that the high variability of parameters such as $F_{max},F_{in},p_{min},p_{max}$ in practice can pose a significant problem when trying to estimate exerted force *F* based on touch pressure *p*. In contrast to the e-skin sensors’ measurement nonlinearity, this cannot be compensated for using different fitting characteristics.

**Figure 15:**
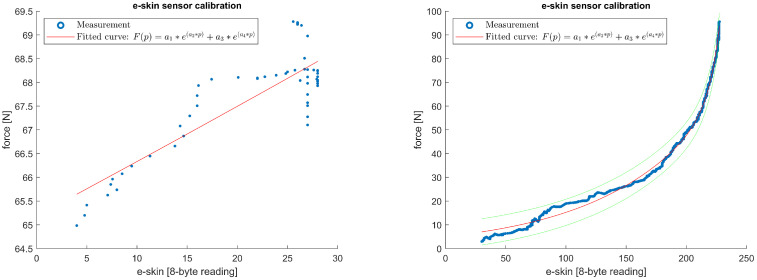
Calibration characteristics fitting problems—comparison of vastly different sensor calibration data. On the left: sensor from row 7, column 26; with force *F* range from 64.9830 N to 69.2800 N; fit parameters ARS=0.7233,RMSE=0.5666. On the right: sensor from row 16, column 30; with force *F* range from 2.9490 N to 95.5800 N; fit parameters ARS=0.9961,RMSE=1.7822.

Finally, it is difficult to define one specific characteristic for all sensors because it varies significantly from sensor to sensor, as shown in Figure 15. Each sensor should have its own parameters for the selected set of functions, e.g., according to Equation (1). Similarly, it is difficult to establish starting points for algorithms that optimise curve fitting parameters for a given set of functions. In the work performed, an invariant group of starting points was experimentally established and used for all curves of one family of characteristics.

In terms of time analysis, data from the e-skin sensors are collected with a delay of about 21 ms. In order to use these measurements for emergency stopping of the robot in response to human touch, it is necessary to increase this speed probably to several ms and to ensure higher reliability of measurement acquisition. The safety of working with a robot should be combined with an analysis of the robot’s control in terms of, e.g., achievable speeds and the possible acceleration and distance of stopping of the manipulator.

Regarding the execution time of the calibration procedure, stage I of this procedure for one sensor for the load and unload phases total is about 80 s. Acquisition of measurements for all 180 tested sensors was approximately 4 h. The long measurement acquisition time is directly caused by the measurement acquisition time of e-skin and Hex-e. Both the e-skin and the reference Hex-e sensor have a limited measurement acquisition frequency. A significant number of measurement points need to be acquired in order to carry out calibration curve fitting. With too few of them, the fit of the model curve to the measurement data can be unreliable. For this purpose, the measurement acquisition time for one e-skin sensor was selected experimentally but can be further optimised as a part of the extension study.

## 4. Conclusions

Solving the problem of calibrating multiple e-skin sensors when their parameters are heterogeneous is of great importance in the current development of human–robot interfaces. The manuscript describes a newly developed robotic procedure for the calibration of touch sensor arrays. Within the presented calibration procedure, it is possible to mitigate the inhomogeneity of e-skin sensors by using a matched model (1) to estimate exerted force values from touch pressure sensor measurements for individually defined parameters. This is represented by substantial variability in parameters such as maximum exerted force, exerted force insensitivity threshold, or range of measured touch pressure for sensors on the e-skin surface. A significant advantage of the presented procedure is the considerable automation—the full process of acquiring measurement data to calibrate only a subset of the e-skin sensors takes approximately 4 h. Thanks to the proposed robotic procedure, a human did not have to deal with this tedious task.

Stage II of the calibration procedure, i.e., the analysis of the measurement data, particularly regarding the selection of the starting points of the algorithms for optimising the parameters of the fitted curves, requires significant human input. Our experience suggests that the starting parameters are best determined in a relationship with the statistics describing the collected measurements of exerted force and touch pressure, e.g., force insensitivity threshold or range of measured touch pressure. Closer investigation in this area may lead to the identification of specific relationships. In the course of the analysis, it was not possible to establish a curve having a specific interpretation related to the physical phenomenon describing the performance of the e-skin sensors. Establishing such a characteristic and relating its parameters to, e.g., force insensitivity threshold or range of measured touch pressure could significantly facilitate calibration step II, i.e., the analysis of the measurement data.

Finally, it is worth considering how the calibration of the exerted force and touch pressure relationships can be simplified. The experience gained from the work on the presented calibration procedure leads to the conclusion that a promising direction for further research could be the self-calibration of the e-skin relative to the robot, without presenting the force standard as a Hex-e device. Self-calibration could, for example, lead to the discovery of a relationship between the control moments in the manipulator joints and the e-skin measurements.

## Figures and Tables

**Table 1:** Goodness of fit statistic summary.

Curve	Phase	Mean ARS	STD ARS	Mean RMSE	STD RMSE
(1)	load	0.9933	0.0248	0.9197	0.5192
	unload	0.9895	0.0280	1.4683	1.0251
(2)	load	0.9774	0.0433	7.0709	2.4793
	unload	0.9836	0.0234	6.3137	2.6965

**Table 2:** Fitting curve coefficients statistics.

Curve (1), Load			Curve (1), Unload			Curve (2), Load			Curve (2), Unload		
$a_{1}$	mean	−2737.93	$a_{1}$	mean	−7681.02	$a_{1}$	mean	202.0638	$a_{1}$	mean	205.6332
	STD	27738.85		STD	61259.33		STD	38.456		STD	40.5454
$a_{2}$	mean	−0.1736	$a_{2}$	mean	−2.2506	$a_{2}$	mean	0.16386	$a_{2}$	mean	0.15937
	STD	2.1728		STD	25.4543		STD	0.1444		STD	0.14263
$a_{3}$	mean	1.2999	$a_{3}$	mean	2.0864	$a_{3}$	mean	33.0249	$a_{3}$	mean	32.5599
	STD	11.5815		STD	10.1773		STD	17.5376		STD	17.9087
$a_{4}$	mean	−0.1159	$a_{4}$	mean	−0.0994						
	STD	1.2163		STD	1.1641						
samples	149		samples	149		samples	149		samples	149	

**Table 3:** E-skin sensors insensitivity threshold $F_{in}$ statistics.

Phase	Mean $F_{in}$	STD $F_{in}$
load	21.3475	15.8522
unload	20.5055	16.2352

## Data Availability

The raw data presented in this study are available in this article Supplementary Materials.

## Supplementary Materials

The following supporting information can be downloaded at: https://www.mdpi.com/article/10.3390/s22166122/s1.
